# Supplementary material for: Antitumor Activity of Nanoparticles Loaded with PHT-427, a Novel AKT/PDK1 Inhibitor, for the Treatment of Head and Neck Squamous Cell Carcinoma
Source: Pharmaceutics. 2021 Aug 12;13(8):1242. doi: 10.3390/pharmaceutics13081242 (PMC8401941; doi:10.3390/pharmaceutics13081242)
Supplement: Supplementary file 1 [file pharmaceutics-13-01242-s001.zip › pharmaceutics-1285326-supplementary.pdf]

pharmaceutics

MDPI

Supplementary Materials: Antitumor Activity of Nanoparticles Loaded with PHT-427, a Novel AKT/PDK1 Inhibitor, for the Treatment of Head and Neck Squamous Cell Carcinoma

Joaquín Yanes-Díaz, Raquel Palao-Suay, María Rosa Aguilar, Juan Ignacio Riestra-Ayora, Antonio Ferruelo-Alonso, Luis Rojo del Olmo, Blanca Vázquez-Lasa, Ricardo Sanz-Fernández and Carolina Sánchez-Rodríguez

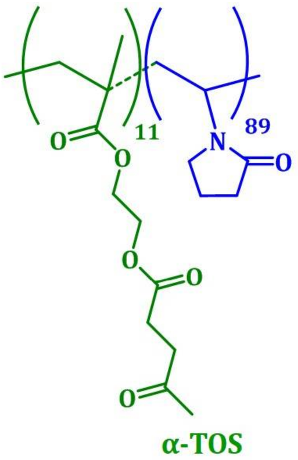

MTOS11

Copolymer molar fraction, yield of the copolymerization reaction, molecular weights, polydispersity (M_w/M_n), and T_g of MTOS 11

f(MTOS) copol	Yield (%)	$M_w \cdot 10^{-3}$	$M_n \cdot 10^{-3}$	M_w/M_n	Tg_1 (ºC)	Tg_2 (ºC)
0.11	79	55.6	30.5	1.8	66	144

α-TOS:

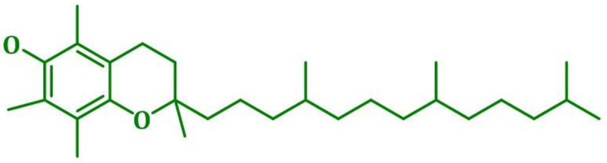

Figure S1. Summary of MTOS11 characterization.

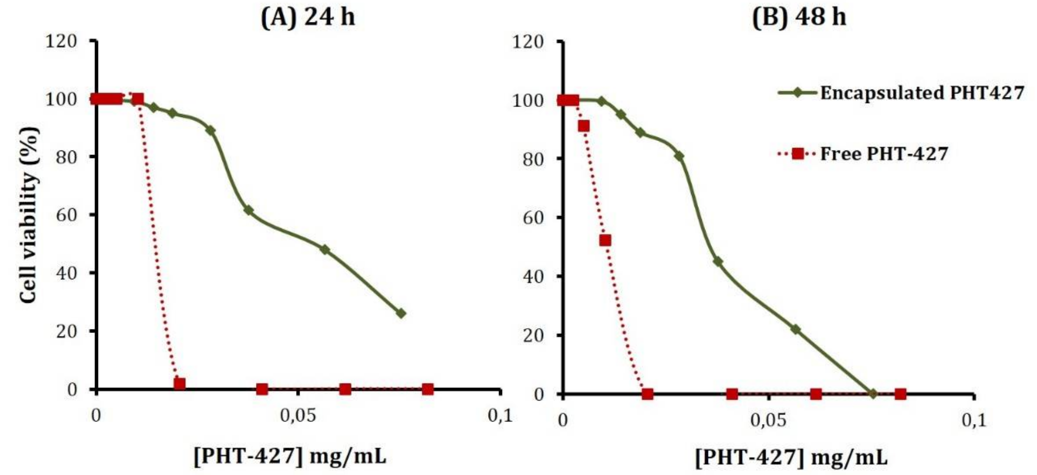

Figure S2. FaDu viability represented against the encapsulated PHT-427 concentration for each NP-427 concentration and compared to free PHT-427 after 24 (**A**) and 48 h (**B**).

Table S1. FaDu cell viability IC50 values of PHT-427, NP-Ø and NP-PHT427 after 24 and 48 of treatment.

Time (h)	Free PHT-427 (mg/mL)	NP-Ø (mg/mL)	NP-PHT427	
			NP-PHT427 (mg/mL)	Encapsulated PHT-427 (mg/mL)
24	0.016	0.738	0.629	0.047
48	0.011	0.735	0.538	0.040
